# *TP53* mutations in Romanian patients with colorectal cancer

**DOI:** 10.1186/s41021-023-00277-2

**Published:** 2023-07-01

**Authors:** Felix Manirakiza, Hidetaka Yamada, Yuji Iwashita, Keiko Ishino, Rei Ishikawa, Zsolt Kovacs, Eva Osvath, Augustin Nzitakera, Simona Gurzu, Haruhiko Sugimura

**Affiliations:** 1 Department of Tumor Pathology, Hamamatsu University School of Medicine, 1-20-1 Handayama, Higashi-Ku, Shizuoka, 431-3192 Japan; 2 Department of Pathology, School of Medicine and Pharmacy, College of Medicine and Health Sciences, University of Rwanda, P.O. Box 3286, Kigali, Rwanda; 3 Research Center of Oncopathology and Translational Research (CCOMT), George Emil Palade University of Medicine, Pharmacy, Science and Technology, Targu-Mures, 540139 Romania; 4 Department of Pathology, George Emil Palade University of Medicine, Pharmacy, Science and Technology, Targu-Mures, 540139 Romania; 5 Department of Biomedical Laboratory Sciences, School of Health Sciences, College of Medicine and Health Sciences, University of Rwanda, P.O. Box 3286, Kigali, Rwanda; 6 Sasaki Institute, Sasaki Foundation, 2-2, Kanda Surugadai, Chiyoda-ku, Tokyo, 101-0062 Japan

**Keywords:** *TP53*, Novel mutation, Romania, Cancer, Tumor suppressor, Genome, Environment, Life style in East Europe

## Abstract

**Background:**

Colorectal cancer (CRC) has been ranked as the second most deadly cancer and the third most diagnosed cancer cases for the year 2020. Specifically for Romania, the number of CRC-related deaths in 2019 was estimated at 6307 people, with a standardized mortality rate of 33.8 per 100,000 inhabitants. Although the tumor protein 53 (*TP53*) gene is intensively studied, there are few data on *TP53* mutations in Romanian CRC. Furthermore, since genetic alterations may show geographical differences, our study aimed to analyze the clinical status and *TP53* somatic variation in Romanian CRC patients.

**Subjects and methods:**

DNA from 40 randomly selected cases of CRC was extracted from formalin-fixed paraffin-embedded tissues and sequenced using direct Sanger sequencing techniques, and variants were annotated according to the recommendations of the Human Genome Variation Society. Novel variants were analyzed using MutationTaster2021 to predict their effects.

**Results:**

The mean age was 63.6 years (range 33–85 years) with a male to female ratio of 2.3. More than 45% (18/40) had an advanced cancer stage (≥ stage III). Mutations were found in 21/40 cases (52.5%), with one case having two mutations, giving a total of twenty-two mutations in the *TP53* coding DNA. These mutations include 3 (13.6%) insertion-deletion mutations, two of which are novel frameshift mutations: c.165delT (in exon 4) and c.928_935dup (in exon 9), both of which are predicted to lead to nonsense-mediated mRNA decay and are classified as deleterious. The remaining 19 (86.36%) were substitution mutations: 1 nonsense and 18 (81.8%) missense mutations, with G > A (n = 7/19; 36.8%) and C > T (n = 6/19; 31.5%) transitions being the most common. The G > T transversion was found in 21.05% (4/19) of the substitution mutations.

**Conclusion:**

We have described two novel frameshift mutations in *TP53*. The discovery of novel mutations following the efforts of The Cancer Genome Atlas and other large-scale cancer genome sequencing projects may be further evidence of the heterogeneous nature of mutations in cancer and may indicate that the identification of carcinogenic mutations is not yet saturated. Further sequencing is therefore needed, especially in less studied populations. Importantly, consideration of their geographical environment will shed light on population-specific carcinogenesis.

**Supplementary Information:**

The online version contains supplementary material available at 10.1186/s41021-023-00277-2.

## Introduction

In the 2020 GLOBOCAN report on cancer statistics, the colorectal cancer (CRC) was ranked as the second most deadly cancer after lung cancer and the third most diagnosed cancer after breast and lung cancer [1]. The incidence of CRC varies widely according to geographical location and socioeconomic status [2, 3]. Incidence rates are approximately four times higher in developed countries than in developing countries [1]. On the other hand, a little variation in mortality rates is observed across different countries because of increased fatality in developing countries [1]. The highest incidence rates for CRC are recorded in European regions, Australia/ New Zealand, and North America. The same trend exists for rectal cancer incidence rates, but the highest rates are found in East Asia [1].

Specifically for Romania, a Southeastern European country, the number of CRC related deaths in 2019 was estimated at 6307 people, with a standardized mortality rate of 33.8 per 100,000 inhabitants [4]. The number of cases is gradually increasing and, due to the lack of screening programs, most cases are still diagnosed at an advanced stage.

Cancer genetics is a key to both prevention and treatment of this deadly disease, but cancer genetics can exhibit geographical differences that may be due to differences in germline variants secondary to founder mutation (s) and/or somatic mutations that are more influenced by environmental carcinogenesis.

The *tumor protein 53* (*TP53*) gene is a key gene in most of human cancers and has been called the “guardian of the genome” due to its role in responding to DNA damage to prevent the spread of damaged DNA [5, 6]. This gene is a tumor suppressor gene, with approximately twelve TP53 isoform proteins currently described [7, 8]. Its proteins act as transcription factors and play many roles in maintaining cell life and integrity, including regulation of cell metabolism, cell cycle, apoptosis, senescence, and DNA damage repair among others [6, 9, 10].

*TP53* is among the mostly studied genes [10], and as more studies dig deeper to learn more about it, more knowledge is being discovered, including novel mutations, single nucleotide polymorphisms (SNPs) and their implication in the development, progression and treatment of various diseases [11–15].

Mutations and/or allelic variants in the *TP53* gene have been proven to influence carcinogenesis, disease progression and/or response to treatment in several cancers including CRC [12, 13, 16]. The commonest *TP53* mutations in different cancers are missense mutations, accounting for approximately 40%, followed by frameshift deletions in 20% and frameshift insertions in 10% [17].

In CRC, the frequency of *TP53* mutations ranges from 33 to 60% [16, 18] but there is insufficiency of available data regarding the *TP53* mutations in Romanian patients with CRC. Additionally, while dietary and environmental factors have been linked with CRC in humans and animals [19–21], recent studies have documented a shift from the typical traditional Romanian diet to other dietary patterns, including a high meat/ high fat and Western patterns [22]. Furthermore, Romanian patients with CRC had a dietary habit linked with a Western style diet [23], and Gavrilaș et al. [23] reported that consumption of processed meat was associated with a sixfold risk (odd ratio of 6, p < 0.001) of developing colon cancer in Romanians.

Given the increasing CRC incidence in Romanians, and the recent change in their diet, our study aimed to analyze the *TP53* mutations in Romanian CRC patients. Moreover, novel somatic mutations in understudied populations would expand our knowledge about *TP53* mutations and CRC carcinogenesis which may be influenced by the dietary pattern of the population and other environmental factors. Therefore, in this paper, we also describe two novel *TP53* frameshift (FS) mutations.

## Materials and methods

### Patients

Forty patients with histologically confirmed CRC were included in this study. Cases were retrospectively collected among patients who benefited surgical resection for a CRC during 2016/2017. The randomly selected cases were histologically confirmed in the Department of Pathology of Clinical Emergency Hospital of Targu-Mures, Romania. All cases included in this study were naïve for preoperative oncologic therapy and all were microsatellite stable.

### DNA extraction

DNA was extracted from formalin-fixed paraffin-embedded (FFPE) tissue blocks using QIAamp® DNA FFPE Tissue Kit (Qiagen GmbH, Qiagen strasse1, 40,724 Hilden, Germany) according to manufacturer’s guidelines. The extracted DNA was quantified using nanodrop® 1000 (ThermoFisher Scientific, Wilmington, CO, USA) spectrophotometer and for all cases 260/280 ratio was between 1.7 and 1.9.

### DNA amplification and sequencing

DNA from exons number 2 to 11 of *TP53* [24] was amplified using HotStarTaq (Qiagen) and sequenced in the forward and reverse directions by the Sanger sequencing method using the BigDye Terminator Cycle Sequencing Reaction Kit, ver.3.1 and ABI 3130xL Genetic Analyzer (ThermoFisher Scientific) as described by Natsume et al. [25].

### Mutation detection, annotation and in silico analysis

DNA sequence data were aligned to *TP53* genomic sequence NC_000017.11 (GRCh38.14) on chr17:7668421–7687490 [26] using both UniproUGENE V.35 [27] and GENETYX® software package ver.14.1.0 (Genetyx Corporation, Tokyo, Japan). Known variants were annotated using dbSNP (build 156) database and following the Human Genome Variation Society (HGVS)’ recommendations. The novel variants were analyzed using MutationTaster2021 [28] in silico tool to predict the consequence of DNA variation. Every detected mutation was confirmed in two independent Sanger sequencing experiments. We only reported new mutations and mutations that are reported to be pathogenic or likely pathogenic or those with MAF < 0.0001 in major databases (ALFA, ClinVar, dbSNP, VariSome, 1000 genome project and TOPMed) (Supplementary Table S1). We did not determine if mutations were purely somatic or germline.

### Ethical approval

This study was approved by the Ethics Committee of the University of Medicine and Pharmacy of Targu-Mures, Romania (agreement no 124/28.07.2016) and by the Ethics Committee of Hamamatsu University School of Medicine (EC HUSM number: 20 − 011).

## Results

Forty patients with a mean age of 63.6 years [range, 33–85 years] and a male/female ratio of 2.3 were included in the database. More than 45% (18/40) had advanced cancer (≥ stage III). Most cases (31/40) were diagnosed in the distal colon (Table 1). Twenty-one out of 40 cases (52.5%) had a mutation in *TP53*, and there was only one case with 2 different mutations, making a total of 22 mutations. Of the 22 mutations counted, 18 (81.81%) were in the DNA binding domain, 19 (86.36%) were substitution mutations and 3 (13.6%) were insertion-deletion mutations, two of which are novel mutations to our knowledge.

**Table 1:** Clinical characteristics of patients

No	Age	Sex	Localization	T stage	N stage
1	33	M	Splenic flexure	3	0
2	62	M	Descendent	3	0
3	67	F	Transverse	3	0
4	77	M	Transverse	3	1
5	59	M	Sigmoid	3	0
6	48	M	Transverse	4b	2
7	46	M	Recto-sigmoid	4b	0
8	51	M	Sigmoid	4	1c
9	59	M	Transverse	3	0
10	58	M	Transverse	4b	1b
11	80	M	Recto-sigmoid	4a	1c
12	74	M	Hepatic flexure	3	0
13	62	M	Recto-sigmoid	3	1a
14	74	F	Sigmoid	3	0
15	54	F	Rectum	1a	1
16	38	M	Recto-sigmoid	3	0
17	66	M	Hepatic flexure	3	1a
18	73	F	Descendent	3	0
19	43	F	Sigmoid	3	2a
20	52	F	Rectum	1	0
21	63	M	Rectum	4a	1c
22	48	M	Sigmoid	3	0
23	85	F	Ceacum	3	2
24	64	M	Rectum	1	0
25	59	M	Rectum	3	2
26	71	M	Sigmoid	3	1
27	61	M	Descendent	3	0
28	79	M	Sigmoid	2	0
29	63	M	Descendent	3	1c
30	76	F	Rectum	2	x
31	66	M	Splenic flexure	3	2
32	78	M	Sigmoid	3	0
33	61	M	Splenic flexure	4a	0
34	72	F	Sigmoid	4a	0
35	62	F	Rectum	2	0
36	74	M	Anal canal	4a	1b
37	61	F	Sigmoid	4a	0
38	68	M	Splenic flexure	4a	1b
39	85	F	Sigmoid	4a	0
40	73	M	Transverse	4b	2

### Two novel frameshift mutations in ***TP53***

**Fig. 1:**
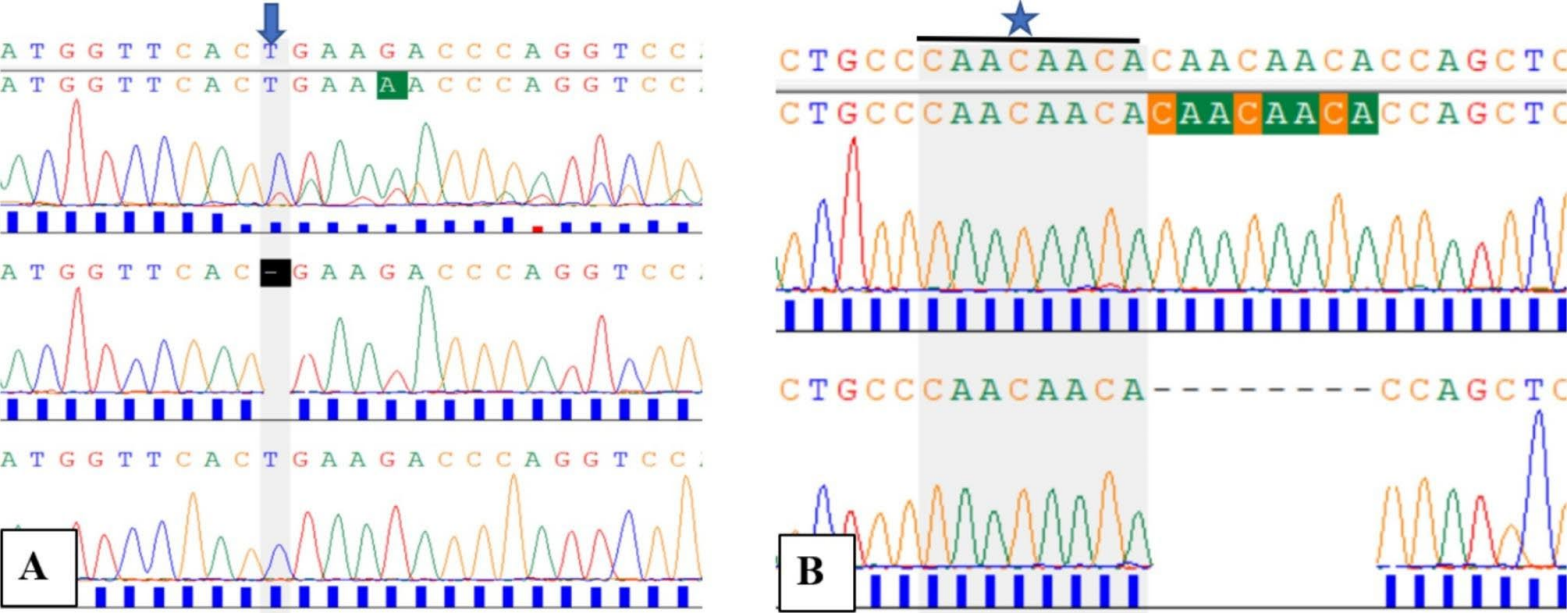
Electropherograms for novel frameshift mutations in Romanian patients with CRC. **A**: c.165delT mutation, the top graph corresponds to case RC21 with mixed electropherogram (see the right side of highlighted area marked by an arrow) secondary to deletion of T. The middle electropherogram shows the same case after cloning of the corresponding PCR product and this cloning allowed to confirm the deletion of T (at the gap space highlighted by a vertical dark column). The lower graph corresponds to a wild type case (a control case without mutation). **B**: c.928_935dup mutation, the top electropherogram corresponds to case RC33 with a sequence of 8 bases that is duplicated; marked by a horizontal black line with a star on top and it was obtained after cloning of its PCR product in plasmid. The lower electropherogram corresponds to a wild type case (a control case without mutation)

In this study we found two novel frameshift mutations, each occurring once: c.165delT which is located in exon 4 and c.928_935dup which is located in exon 9. Their details are shown in Table 2 and their electropherograms are shown in Fig. 1. The wild-type amino acid sequence of the TP53 protein is 341 in length (with the stop codon at position number 342), the deletion mutation c.165delT introduced a premature stop codon at position number 122, so the mutated amino acid sequence was predicted to result in a truncated protein of only 121 amino acids (the stop amino acid at position 122). The novel frameshift duplication mutation c.928_935dup is predicted to be 47 bp shorter than the normal protein. The 2 novel frameshift mutations are predicted to be deleterious by MutationTaster2021 because the mutated amino acid sequences could lead to nonsense-mediated mRNA decay (NMD) and the corresponding protein features would be affected.

**Table 2:** Two novel frameshift TP53 mutations in Romanian patients with colorectal cancer

Sample ID	Sex	Age	Genomic co-ordinate (hg38)	Coding DNA description ^#^	Genotype	Protein description	Exon number	Mutation type	Predicted mutation effect^$^
RC21	M	63	g.7,676,204delA	c.165delT	t/-	p.E56Kfs*67	4-exon	frameshift	Deleterious/disease causing
RC38	M	68	g.7,673,600_7,673,593dup	c.928_935dup	-/aacaacac	p.S313Tfs*35	9-exon	frameshift	Deleterious/disease causing

^#^ Coding DNA is described using NM_000546.6,

^$^ Mutation effect was predicted using MutationTaster2021, this web-based application tool uses Random Forest models to predict the effect of DNA variants and results are provided as tree votes in a binary prediction: deleterious (how many Random Forest decision trees indicate deleteriousness) versus benign (how many decision trees indicate benign change) [28]

### Other ***TP53*** mutations in our study population of Romanian CRC patients

In addition to the novel mutations described above, we found 17 different types of mutations: 1 deletion mutation in one case and 16 substitution mutations, of which the p.R248Q occurred in 3 cases and p.R273C in 2 cases (Table 3). Because of these recurrent mutations, the total number of substitution mutations was 19, of which only one was a nonsense mutation (p.R342*) occurring once in exon 10. The remaining substitutions were missense mutations. Exons 5 and 6 each had 3 different mutations, and 3 mutations occurred in exon 8 (all at the residue p.R273). Exon 7 was the most frequently mutated with 9 mutations, 4 of them at amino acid position R248 (p.R248Q or p.R248W) and another 5 different mutations at different amino acid residues. Further analysis of the 19 substitution mutations showed that G > A (n = 7/19; 36.8%) and C > T (n = 6/19; 31.5%) transitions were the most frequent. The G > T transversion was found in 21.05% (4/19) of the substitution mutations.

**Table 3:** List of previously reported TP53 mutations present in our study population^&^

Sample ID	Genomic co-ordinate (hg38)	Coding DNA description^#^	Protein description	Genotype	Exon number	Effect	Wild type codon	Mutated codon	dbSNP_ID
RC33	g.7,676,106delG	c.267delC	p.S90Pfs*33	c/-	4-exon	frameshift	TCC	N/A	NA^$^
RC28	g.7,675,157G > A	c.455 C > T	p.P152L	c/t	5-exon	missense	CCG	CTG	rs587782705
RC37	g.7,675,125 A > G	c.487T > C	p.Y163H	t/c	5-exon	missense	TAC	CAC	rs786203436
RC31	g.7,675,077G > A	c.535 C > T	p.H179Y	c/t	5-exon	missense	CAT	TAT	rs587780070
RC07	g.7,674,947 A > T	c.584T > A	p.I195N	t/a	6-exon	missense	ATC	AAC	rs760043106
RC02	g.7,674,924 C > T	c.607G > A	p.V203M	g/a	6-exon	missense	GTG	ATG	rs730882003
RC20	g.7,674,887 C > T	c.644G > A	p.S215N	g/a	6-exon	missense	AGT	AAT	rs587782177
RC13	g.7,674,250 C > T	c.713G > A	p.C238Y	g/a	7-exon	missense	TGT	TAT	rs730882005
RC05	g.7,674,238 C > T	c.725G > A	p.C242Y	g/a	7-exon	missense	TGC	TAC	rs121912655
RC29	g.7,674,233 C > A	c.730G > T	p.G244C	g/t	7-exon	missense	GGC	TGC	rs1057519989
RC19	g.7,674,229 C > A	c.734G > T	p.G245V	g/t	7-exon	missense	GGC	GTC	rs121912656
RC27	g.7,674,221G > A	c.742 C > T	p.R248W	c/t	7-exon	missense	CGG	TGG	rs121912651
RC26	g.7,674,220 C > T	c.743G > A	p.R248Q	g/a	7-exon	missense	CGG	CAG	rs11540652
RC32	g.7,674,220 C > T	c.743G > A	p.R248Q	g/a	7-exon	missense	CGG	CAG	rs11540652
RC37	g.7,674,220 C > T	c.743G > A	p.R248Q	g/a	7-exon	missense	CGG	CAG	rs11540652
RC40	g.7,674,216 C > A	c.747G > T	p.R249S	g/t	7-exon	missense	AGG	AGT	rs28934571
RC09	g.7,673,803G > A	c.817 C > T	p.R273C	c/t	8-exon	missense	CGT	TGT	rs121913343
RC18	g.7,673,803G > A	c.817 C > T	p.R273C	c/t	8-exon	missense	CGT	TGT	rs121913343
RC36	g.7,673,802 C > A	c.818G > T	p.R273L	g/t	8-exon	missense	CGT	CTT	rs28934576
RC39	g.7,670,685G > A	c.1024 C > T	p.R342*	c/t	10-exon	nonsense	CGA	TGA	rs730882029

^&^The mutations described in this table have been reported in dbSNP [29], and/or COSMIC [30]. For each mutation, we have included its dbSNP identification number in the dbSNP_ID column

^#^ Coding DNA is described using NM_000546.6, N/A: Not applicable (as there has been frameshift),

NA^$^: In dbSNP (build 156) this mutation is described under rs587783062. It is a deletion (homopolymer) that may be at any position of the range chr17:7,676,102–7,676,106 (GRCh38.p13)

## Discussion

*TP53* mutations were found in 21 out of 40 cases (52.5%). The reported mutation rates in CRC vary from study to study and they range from 33 to 60% [16, 18]. The difference in mutation rates may be explained by the different methods used to analyze mutation status. Mainly, some studies have analyzed exons 5 to 8 or 5 to 9, while others have analyzed exons 2 to 11, but with other possible range of analysis [17, 31].

Previous studies in CRCs from Romanian patients were reported by Murarasu et al. [32], who described 18 different single nucleotide variants (SNVs) in the coding region of *TP53* and 2 SNVs in the intronic region. Only 2 SNVs (NM_000546.6: c.455 C > T and NM_000546.6: c.817 C > T) were identical in both studies. No other reports were found in the literature regarding the spectrum of *TP53* mutations in Romanian patients with CRC. Although our study sample was small, the percentage of transition mutations G > A (36.8%) and C > T (31.5%) was close to that reported in the Catalogue Of Somatic Mutations In Cancer (COSMIC): G > A with 41.49% and C > T with 31.73% for large intestine cancer [30,33].

The novel frameshift mutation c.165delT is a mutation with deletion of T. Mutations with deletion of T are described in the COSMIC v97 Mutational Signatures v3.3 under the insertion-deletion (ID) mutational signature ID18 [34]. The ID18 mutation signature is mainly found in CRC and normal colorectal epithelial cells [33–36]. The proposed etiology for this signature is exposure to colibactin, a genotoxic compound produced by *E.coli* bacteria bearing *pks* pathogenicity island [34, 36].

Furthermore, in one of our patients we found a frameshift mutation c.267delC which has been previously reported in ClinVar [37], Medical Genomics Japan Variant Database (MGeND) [38] and in the COSMIC [39] as a germline [37] and as a somatic mutation in various cancers [39]. This is a homopolymer deletion mutation, with four additional equivalent representations and has been predicted to be pathogenic [40, 41]. This frameshift deletion mutation will result in a truncated amino acid sequence of the same length as the novel frameshift deletion mutation (c.165delT) described above. For both of them, the last 33 amino acids sequence were predicted to be identical and their mutated amino acid sequences were predicted to lead to an NMD. From this perspective of some similarity between the two frameshift mutations, we can expect that the novel frameshift mutation c.165delT would also be a potentially disease-causing mutation.

The nonsense mutation c.1024 C > T (p.R342*) leads to a protein truncation of 52 amino acids and is predicted to cause NMD and loss of many protein features. This mutation is reported and interpreted as pathogenic in ClinVar (ID: 182,970) for several conditions (Li- Fraumeni syndrome, ovarian neoplasms, gallbladder cancer, hereditary cancer predisposition syndrome and colonic diverticular disease) [42]. It has been found in both somatic and germline conditions. However, it is not reported in 1000G, ExAC and gnomAD. On the other hand, it is reported in the top 100 cancer driver mutations and accounts for 0.33% of all cancer patients [43].

As stated above, all of our cases with nonsense and frameshift mutations are predicted to lead to NMD. In vivo, NMD has been described as a complex surveillance process [44] with cellular variability in its efficiency [45, 46], and there is a possibility that mRNA with a premature stop codon may still not be degraded via NMD, leading to the expression of a truncated protein [47, 48].

However, it should be noted that since NMD has been predicted by in-silico tools for mutations detected in cancer tissues, therefore we can expect that the tumor tissues may still express the TP53 protein from wild-type transcripts. TP53 protein levels and *TP53*-mediated transactivation are negatively regulated by mouse double minute 2 (MDM2) [48–51]. Moreover, previous studies have shown that mutant p53 induces the stabilization of MDM2 [52, 53] and MDM2 amplification has been reported in CRC including in some cases of mutant TP53 CRC [54]. Therefore, we might expect that the level of wild-type TP53 protein would be strongly affected by the corresponding level of MDM2 expression in the same tumor.

Most of the missense SNVs in the *TP53* gene are located in hotspot mutation positions. Eighteen out of twenty-two (81.8%) are missense mutations and are all located in the DNA binding domain of the TP53 protein (spanning from position 102–292 amino acids), which is a common occurrence in cancer cells [55]. When considering the top most frequently mutated residues in the TP53 protein, we found that 14/18 (77.7%) of the missense mutations in our study are recorded in the top 50 most frequently mutated residues and 9/18 (50%) are in the top 10 hotspot mutation positions [55]. These hotspot positions are: R248, R273, R249 and G245 with 4, 3, 1 and 1 cases respectively. Murarasu et al. [32] found that mutations in 4 hotspots (R175, G245, R273 and R282) accounted for 48.3% in their study of Romanian CRC cases. Finally, note that all, except three (V203, S215 and G244) of the missense mutations residue in this study are recoded among the top 100 cancer driver mutations of human cancers [43].

The mechanisms by which mutations in the *TP53* gene may contribute to cancer pathogenesis and progression have evolved and will continue to evolve over time. For example, Wills et al. [56] found that mutant p53 exerts a dominant-negative effect compared to wild-type by suppressing both the DNA binding ability, the potential to induce cell cycle arrest and the growth suppressive utility of wild-type TP53. Stabilization and accumulation of MDM2 induced by different mutant TP53 cancer cells [52, 53] and amplification of MDM2 in mutant TP53 CRC have been reported. We can hypothesize that there may be a combined effect of the degradation of wild-type TP53 protein by MDM2 and the dominant negative effect of mutant TP53 in favor of cancer development and/or progression. This hypothesis needs to be evaluated using in vitro and in vivo strategies.

### Limitations of the study

Our study has some limitations that further research may be able to address. We only analysed tissue samples from patients, without testing family members. Hence, we could not confirm if the mutations were germline or purely somatic. The functional analysis of the protein from cases with novel mutations that we describe in this paper was done using an in silico tool. Therefore, the in vivo or in vitro nature of the TP53 protein should be investigated, as there may be some differences between what is predicted by the computer tools and what might occur under natural conditions in terms of the type and expression level of the *TP53* transcripts and protein. Our data are too small to give any insight that the mutations in CRC in Romania may reflect known or unknown specific environmental or dietary exposures, but the accumulation of the mutation spectrum would highlight the risk and the reason for the increase of CRC in Romania in the future. Recently, we observed the difference in *TP53* mutation spectrum between Eastern Europe and East Asia [25], and it would be interesting to see the status of *TP53* mutation in CRC in East Asia for comparison.

## Conclusion

In this study, we found that most of *TP53* missense mutations are concentrated in the DNA binding domain, as it has been previously reported. However, we identified two novel frameshift mutations, both of which are predicted to be deleterious/ disease causing mutations and will require further studies to confirm their functional consequences in vivo. Our findings highlight the heterogeneous nature of the mutational status within cancers. The continued discovery of new mutations after the efforts of The Cancer Genome Atlas and other large-scale cancer genome study projects may indicate that the collection of carcinogenic mutations that vary from individual to individual is not yet saturated. Further sequencing is therefore needed, especially in geographical populations that have not yet been studied. We can expect that more new mutations or variants will continue to be discovered, especially in understudied populations, and that consideration of their geographical environment will shed light on population-specific carcinogenesis.

## Electronic supplementary material

Below is the link to the electronic supplementary material.

Supplementary Material 1

## Data Availability

The data that support the findings of this study are available from the corresponding author upon reasonable request.

## List of abbreviations

ALFA: Allele frequency aggregator
COSMIC: Catalogue of somatic mutations in cancer
CRC: Colorectal cancer
dbSNP: Database of single nucleotide polymorphism.
DNA: Deoxyribonucleic acid
FFPE: Formalin-fixed paraffin-embedded
FS: Frameshift
gnomAD: Genome Aggregation Database
GRCh: Genome Research Consortium human build
MDM2: Mouse double minute 2 or MDM2 proto-oncogene
MGeND: Medical Genomics Japan Variant Database
NMD: Nonsense-mediated mRNA decay
RNA: Ribo Nucleic Acid
SNP: Single Nucleotide Polymorphism
SNV: Single Nucleotide Variant
TOPMed: Trans-omics for precision medicine
*TP53*: Tumor Protein 53
